# Tracking the Details of an Immune Cell Rendezvous in 3-D

**DOI:** 10.1371/journal.pbio.0030206

**Published:** 2005-05-03

If you stopped to consider the potentially pathogenic encounters waiting outside your door on any given day, you might never leave home. Then you would just have to contend with the billions of microbes reproducing on your toothbrush and kitchen sponge. Of course, most of us safely navigate our microbe-filled world thanks to an immune system that manufactures billions of lymphocytes a day, in case any of those microbes proves malicious.

Lymphocytes arise in the bone marrow, where B lymphocytes mature; T lymphocyte precursors migrate to the thymus to mature. Both T and B cells then travel to distinct regions in the spleen and lymph nodes—B cells amassing in follicles and T cells in T zones—in search of alien antigens. After encounters with antigen, B and T lymphocytes migrate to the edges of their respective zones and compare notes. These B cell–helper T cell interactions are essential for an effective B cell–mediated antibody response. One would not expect such crucial interactions to be left to chance alone, but evidence of directed migration has not been generated—until now.

In a new study, Takaharu Okada and Jason Cyster at the University of California at San Francisco together with Mark Miller and Mike Cahalan at the University of California at Irvine and several colleagues used a groundbreaking technology called two-photon microscopy to visually inspect intact lymph nodes extracted from mice to investigate this lymphocyte rendezvous. They discovered a combination of random and directed behaviors: antigen-engaged B cells move randomly along the follicle outskirts, then undergo directed migration near the follicle/T-zone border as they home in on their helper T counterparts. (For more on two-photon microscopy, see the Primer by David Piston [10.1371/journal.pbio.0030207] and “Tracking Migrating T Cells in Real Time” [DOI: 10.1371/journal.pbio.0030205].)

Once a B cell recognizes an antigen via immunoglobulin receptors on its surface, it will not proliferate, differentiate into a plasma cell, and generate mass quantities of antibodies without the go-ahead from a helper T cell. To investigate the dynamics of this process, Okada, Miller, and colleagues transferred fluorescently labeled HEL-specific transgenic B cells, or Ig-tg B cells (engineered to secrete antibodies against the model antigen, hen egg lysozome [HEL]), and nonaltered B cells into mice with identical genetic backgrounds. An hour after HEL injections, lymph nodes were removed from the mice for microscopic analysis. The Ig-tg B cells were “fully occupied” by HEL antigen and—unlike the naïve (unengaged) non-Ig-tg cells—had begun to aggregate along the edge of the follicles.

After antigen binding, the Ig-tg cells grew sluggish compared to naïve cells, then headed for the B-zone/T-zone (B/T) border. Half of the primed cells reached the B/T boundary, compared to 20% of the naïve cells, and did so by (mostly) taking a path that was closer to a straight line—a sign of directed migration. Ratios of path length plotted against displacement from the path show that antigen-engaged B cells tacked toward the boundary when they got within about 140 microns of it.[Fig pbio-0030206-g001]

**Figure pbio-0030206-g001:**
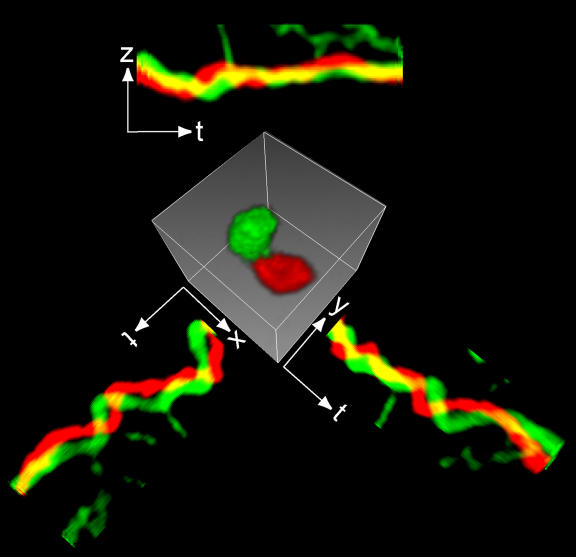
Two-photon microscopy reveals the three-dimensional dynamics of B cell (red) and T cell (green) conjugate within lymph tissue in real time

The authors go on to show that antigen-engaged B cells need the chemokine receptor CCR7 to follow directions to the T zone—which contains an abundant supply of CCR7's signaling protein, or ligand—CCL21. Besides concentrating in the T zone, CCL21 also showed up in follicles, in an increasing gradient from the follicle periphery to the boundary. Okada, Miller, and colleagues studied interactions between antigen-engaged B cells and activated helper T cells in a transgenic mouse model and found that only B and T cells with cognate antigens formed stable pairs, which moved at the B cells' discretion.

Based on these findings, the authors conclude that once antigen-engaged, B cells follow the long-range chemokine gradient to the B/T boundary. After arrival at the boundary the B cells can undergo multiple, even polygamous, contacts with T cells—which might facilitate optimal pairings—before B cell proliferation and antibody production begins. Whether promiscuous and monogamous liaisons produce different B cell reactions is unclear. And though CCR7 helps B cells find the border, it's not clear what keeps them there. But thanks to the T lymphocyte–B lymphocyte dynamics outlined here, immunologists have plenty of avenues for exploring these questions to further elucidate the complex interactions underlying an effective antibody attack.

